# Prenatal Exposure to Lipopolysaccharide Results in Myocardial Fibrosis in Rat Offspring

**DOI:** 10.3390/ijms160510986

**Published:** 2015-05-14

**Authors:** Xin Chen, Yujie Tang, Meng Gao, Shugang Qin, Jianzhi Zhou, Xiaohui Li

**Affiliations:** 1 Institute of Materia Medical, College of Pharmacy, Third Military Medical University, Chongqing 400038, China; E-Mails: kaoyanchenxin@163.com (X.C.); gm0716@126.com (M.G.); qsg18323213193@163.com (S.Q.); 2 Department of Pharmacy, Xiangyang Central Hospital, Xiangyang 441021, China; E-Mail: tyjylx@163.com

**Keywords:** myocardial fibrosis, lipopolysaccharide, pyrrolidine dithiocarbamic acid, pregnant

## Abstract

The epigenetic plasticity hypothesis indicates that exposure during pregnancy may cause adult-onset disorders, including hypertension, myocardial infarction and heart failure. Moreover, myocardial fibrosis coincides with hypertension, myocardial infarction and heart failure. This study was designed to investigate the effects of prenatal exposure to lipopolysaccharide (LPS) on myocardial fibrosis. The result showed that at six and 16 weeks of age, the LPS-treated offspring exhibited increased collagen synthesis, an elevated cardiac index (CI), higher mRNA levels of TIMP-2 and TGFβ and a reduced mRNA level of MMP2. The protein levels corresponded to the mRNA levels. The offspring that were prenatally treated with pyrrolidine dithiocarbamic acid (PDTC), an inhibitor of NF-κB, displayed improvements in the CI and in collagen synthesis. Moreover, PDTC ameliorated the expression of cytokines and proteins associated with myocardial fibrosis. The results showed that maternal inflammation can induce myocardial fibrosis in offspring during aging accompanied by an imbalance of TIMP-2/MMP2 and TGFβ expression.

## 1. Introduction

Cardiovascular disease is a major disease that is harmful to human health. Most cardiovascular diseases are accompanied by myocardial remodeling, and cardiac fibrosis is an important part of myocardial structural remodeling. Myocardial fibrosis (MF) often occurs during the progression of rheumatic heart disease, myocardial remodeling during hypertensive heart disease and other diseases [1]. MF results from disruption of the equilibrium between the synthesis and degradation of collagen, which leads to an excessive accumulation of collagen fibers in the myocardium [2]. The pathogenesis of MF is not completely clear; its regulation involves the extracellular matrix (ECM) system [3], the renin-angiotensin aldosterone system [4,5], a variety of cytokines, cell apoptosis and other systems [6].

Under physiological conditions, cardiac fibroblasts (CFs) are relatively inert cells that maintain myocardial ECM homeostasis. However, in response to cardiac injury or stress, CFs can adopt a specialized myofibroblast phenotype [7]. Myofibroblasts express increased levels of ECM and TGFβ. The ECM is very important for maintaining cardiac structure and function and plays a role in the processes of proliferation and differentiation. The collagens are the most abundant proteins of the ECM and well-accepted tissue markers of cardiac remodeling. The adult myocardium consists of fibrillar collagen type I (85%) and type III (11%). Myocardial ECM remodeling is essentially regulated by pro-fibrotic and anti-fibrotic factors, among them the transforming growth factor-β (TGFβ), which is the best-known and most-studied pro-fibrotic effectors in the heart [8]. At the same time, collagen turnover is regulated by proteolytic enzymes matrix metalloproteinases (MMP) and tissue inhibitors of metalloproteinases (TIMP). The ratio of circulating MMP/TIMP maintains the equilibrium of collagen deposition and degradation within the myocardium [9].

Lipopolysaccharide (LPS) is a strong inducer of innate immunity. Its effects are mediated by its interaction with a member of the Toll-like receptor (TLR) family [10]. In previous studies, we have discovered that following prenatal exposure to LPS, rat offspring suffer from hypertension, cardiomegaly, obesity and Alzheimer’s disease [11,12]. Based on previous reports, this study was designed to explore the effect of prenatal exposure to LPS (0.79 mg/kg) combined with the NF-κB inhibitor pyrrolidine dithiocarbamate (PDTC) on rat offspring. To accomplish this task, MF was evaluated in rat offspring at various time points. Changes in the gene and protein expression levels of TIMP-2, MMP2 and TGFβ were examined to explore the mechanism by which prenatal exposure to LPS induces MF and the role of this mechanism in the development of MF.

## 2. Results

### 2.1. Histological Examination

**Figure 1:**
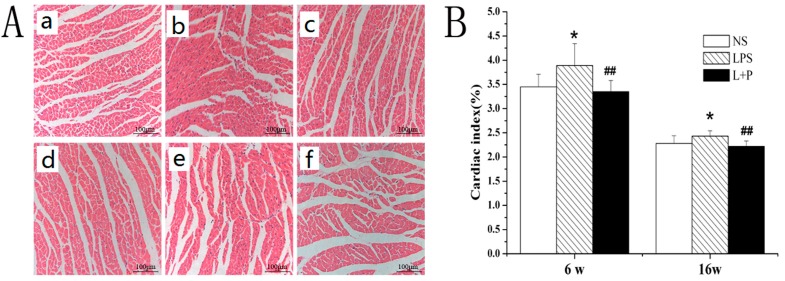
(**A**) Representative photomicrographs show the typical myocardial structure in the various groups (hematoxylin-eosin stain; 200×). (**a**) six-week-old NS-treated rat; (**b**) six-week-old LPS-treated rat; (**c**) six-week-old LPS + pyrrolidine dithiocarbamate (PDTC)-treated rat; (**d**) 16-week-old NS-treated rat; (**e**) 16-week-old LPS-treated rat; (**f**) 16-week-old LPS + PTDC-treated rat. * *p* < 0.05 compared with the control offspring; ^##^ *p* < 0.01 compared with the LPS-treated rat offspring; (**B**) Effect of prenatal exposure to LPS or LPS + PDTC on the cardiac index (CI) evaluated in the offspring.

Compared with the control rats, the cardiac index (CI) was markedly increased in the LPS group at six and 16 weeks of age (*p* < 0.05). All of the indices were significantly decreased in the LPS + PDTC group compared with the LPS group (*p* < 0.01) (Figure 1B).

Using an optical microscope, we observed that the myofibrils of the six- and 16-week-old rat offspring were contiguously aligned in the control rats (Figure 1Aa,d) and that the morphology and structure of the nuclei and cells was normal. In contrast, the cardiomyocytes were hyperplastic; the intercellular substance was expanded; and the myofibrils displayed a disrupted, disordered arrangement in the LPS-treated group (Figure 1Ab,e). Following treatment with LPS and PDTC, the morphology of the myocardium was significantly improved (Figure 1Ac,f). Furthermore, the myocardial fibers were contiguously and more neatly arranged, and the morphology and structure of the nuclei and the cells were normal.

### 2.2. Histopathological Observation of Mouse MF via Sirius Red and Masson Staining

**Figure 2:**
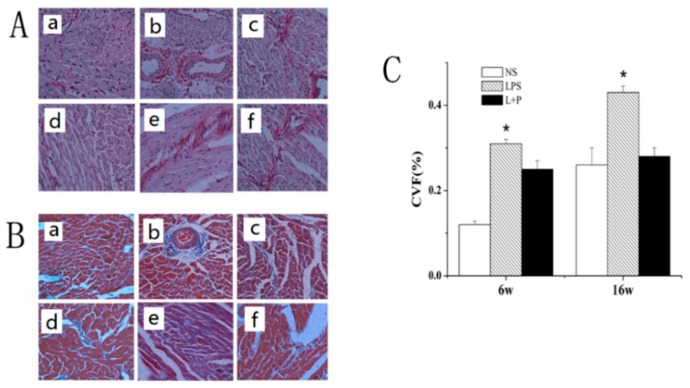
Histopathological changes in the mouse myocardium. (**A**) Sirius red staining (400×): (**a**) six-week-old NS-treated rat; (**b**) six-week-old LPS-treated rat; (**c**) six-week-old LPS + PTDC-treated rat; (**d**) 16-week-old NS-treated rat; (**e**) 16-week-old LPS-treated rat; (**f**) 16-week-old LPS + PTDC-treated rat; (**B**) Masson staining (400×): (**a**) six-week-old NS-treated rat; (**b**) six-week-old LPS-treated rat; (**c**) six-week-old LPS + PTDC-treated rat; (**d**) 16-week-old NS-treated rat; (**e**) 16-week-old LPS-treated rat; (**f**) 16-week-old LPS + PTDC-treated rat. No collagen accumulation was observed in the control group (**A**.**a**, **A**.**d**, **B**.**a**, **B**.**d**); many collagen fibers were observed in the LPS group (**A**.**b**, **A**.**e**, **B**.**b**, **B**.**e**); and few collagen fibers were observed in the LPS + PDTC group (**A**.**c**, **A**.**f**, **B**.**c**, **B**.**f**); (**C**) The collagen volume fraction (CVF), which was calculated by quantitative morphometry using an automated image analysis system. The data are presented as the means ± SD; *n =* 8. * *p* < 0.05 compared with the NS group.

Myocardial collagen expression was observed via Sirius red and Masson staining; the results of collagen staining are shown in Figure 2A,B, and the results of statistical analysis are shown in Figure 2C. Compared with the control group, the collagen protein expression level of the LPS group was significantly increased at six and 16 weeks of age (*p* < 0.01 and *p* < 0.05, respectively). However, the collagen protein expression level was significantly decreased in the LPS + PDTC group compared with the LPS group (*p* < 0.05).

### 2.3. Prenatal Exposure to LPS Influences Expression of the Matrix Metalloproteinases System Components TIMP-2 and MMP-2

**Figure 3:**
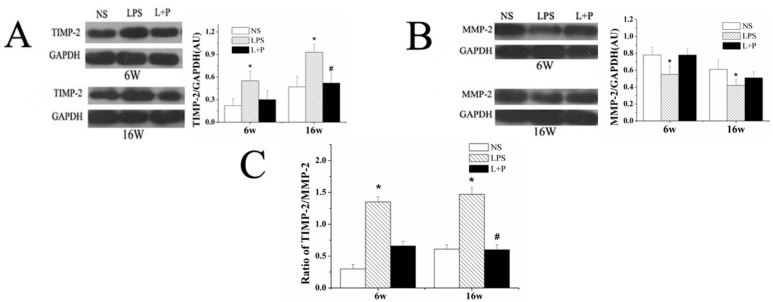
Effects of prenatal exposure to LPS or LPS + PDTC on the expression of TIMP-2 (**A**), MMP2 (**B**) and the TIMP-2/MMP2 ratio (**C**) in heart tissue from six- and 16-week-old rat offspring. The values are presented as the means ± SD. * *p* < 0.05 compared with the NS group; ^#^ *p* < 0.05 compared with the LPS group.

At six and 16 weeks of age, the rats were sacrificed, and protein extracts were prepared from the heart to investigate the expression of TIMP-2 and MMP-2 in the three groups of mice: those injected with i.p. saline (NS), LPS or LPS with PDTC. As shown in Figure 3A,B, TIMP-2 protein expression was significantly higher, but MMP protein expression was significantly lower in the heart tissue from the LPS group than in that from the control group at six and 16 weeks of age. PDTC treatment decreased the expression level of TIMP-2, although this difference was significant only at 16 weeks. Furthermore, PDTC treatment increased the expression of MMP-2, but this difference was not significant. Prenatal exposure to LPS increased the protein expression of TIMP-2 and decreased the expression of MMP-2 in the myocardium. Furthermore, the intraperitoneal administration of PDTC prevented this increase in the TIMP-2/MMP2 ratio (Figure 3C).

### 2.4. Prenatal Exposure to LPS Increased TGFβ-1 and TGFβ-2 Protein Expression

**Figure 4:**
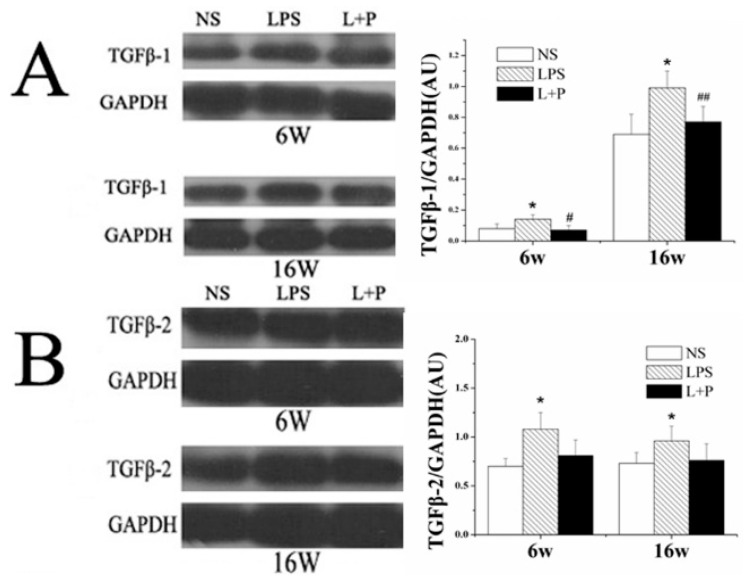
Effects of prenatal exposure to LPS or LPS + PDTC on the expression of TGFβ-1 (**A**) and TGFβ-2 (**B**) in heart tissue from six- and 16-week-old rat offspring. The values are presented as the means ± SD. * *p* < 0.05 compared with the NS group; ^#^ *p* < 0.05, ^##^ *p* < 0.01 compared with the LPS group.

The protein expression levels of TGFβ-1 and TGFβ-2 in the offspring at six and 16 weeks of age were determined via Western blot (Figure 4A,B). Compared with the control group, the protein expression of TGFβ-1 and TGFβ-2 in the LPS group was increased at six and 16 weeks of age (*p* < 0.05). PDTC treatment decreased the expression level of TGFβ-1 (six weeks: *p* < 0.05; 16 weeks: *p* < 0.01) and TGFβ-2, although this difference in TGFβ-2 expression was not significant.

### 2.5. RT-PCR Analysis of mRNAs Related to MF

**Figure 5:**
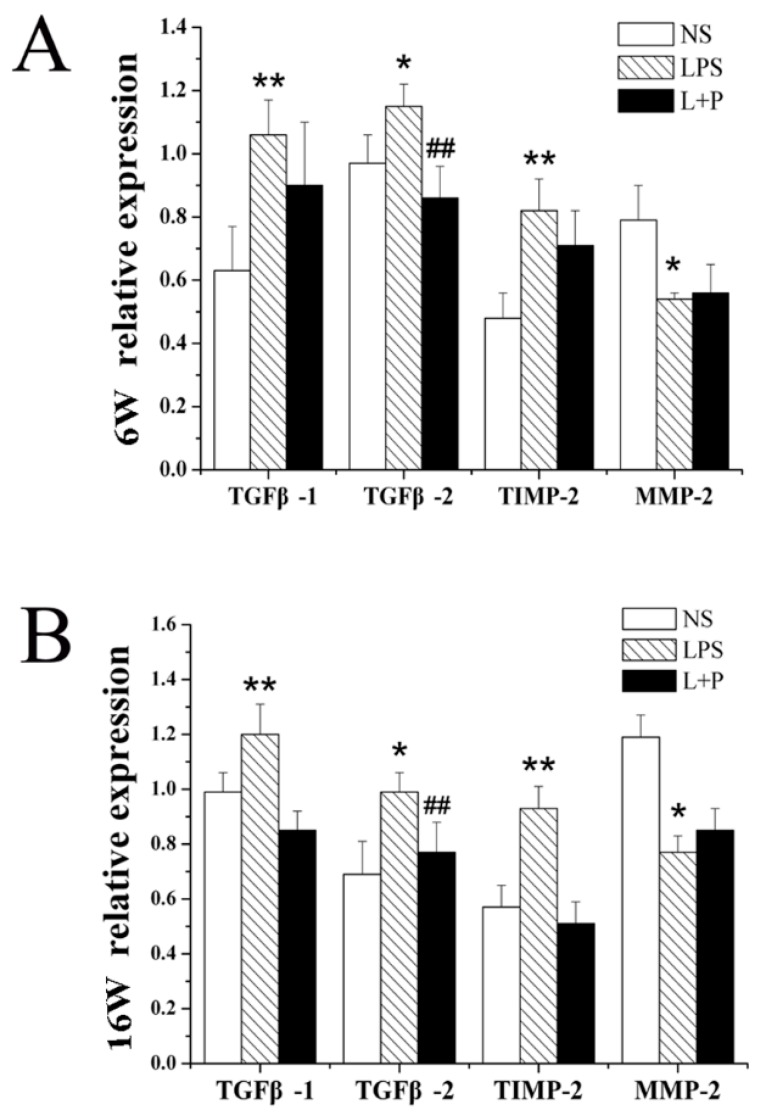
Effects of prenatal exposure to LPS or LPS + PDTC on mRNA expression in heart tissue from rat offspring. The mRNA expression of TGFβ-1, TGFβ-2, TIMP-2 and MMP2 in the heart of six- (**A**) and 16-week-old rats (**B**). *n =* 8. The values are presented as the means ± SD. * *p* < 0.05, ** *p* < 0.01 compared with the NS group; ^##^ *p* < 0.01 compared with the LPS group.

Compared with the NS group, the mRNA expression of TGFβ-1, TGFβ-2 and TIMP-2 in myocardial tissue was significantly increased in the offspring of the LPS group at six and 16 weeks of age. PDTC treatment markedly inhibited these increases in TGFβ-1, TGFβ-2 and TIMP-2 mRNA expression, although only the expression of TGFβ-2 at six weeks of age and the expression of TIMP-2 at 16 weeks of age displayed significant differences. Moreover, the MMP2 mRNA expression levels in the LPS group were lower than those in the NS group at six and 16 weeks of age; and PDTC treatment reduced this change in mRNA expression, although these differences were not significant (Figure 5).

## 3. Discussion

Fibrosis appears as histological changes when the body is damaged or under long-term abnormal stimulation and represents the primary method of organ self-repair [13]. However, fibrosis also provides a material basis for the emergence of certain diseases [4,14]. The responses of the circulatory system to myocardial ischemia, inflammation, aging and other stimuli include increased local myocardial cell apoptosis, increased myocardial fiber proliferation and an increased amount of ECM, of which collagen is the primary component; all of these responses lead to MF [15,16]. Many experimental studies have traced the cause of heart disease back to early life [17,18]. Previous research by our group has revealed that inflammatory stimuli during pregnancy can result in hypertension, obesity and cardiac remodeling in rat offspring. Furthermore, an animal model of prenatal exposure to LPS was successfully established in our laboratory.

In this study, we successfully duplicated the model of prenatal exposure to LPS (on the eight, 10th and 12th gestational days) and found that prenatal exposure to LPS resulted in significant increases in the CI and the myocardial collagen volume in rat offspring at six and 12 weeks of age. The results of the histological observations showed that the cardiomyocytes were hyperplastic, that the intercellular substance was expanded and that the myofibrils displayed a disrupted, disordered arrangement in the LPS group. Myocardial collagen was observed to be significantly increased in the LPS group. All of the above results demonstrated that prenatal exposure to LPS induced MF in the rat offspring. However, PDTC reduced the degree of MF, thereby improving cardiac function by inhibiting TGFβ-1, TGFβ-2 and TIMP-2 protein expression.

The ECM in the heart plays an important role in the maintenance of cardiac structure and function [19]. ECM remodeling causes myocardial fibrosis and progressive ventricular expansion, ultimately leading to heart failure. MMPs in the myocardium, which can degrade almost all ECM in the heart, are the primary mediators of substrate degradation during myocardial remodeling [20,21]. MMPs can be regulated at the transcription level and by the endogenous physiological TIMPs. Therefore, the level of MMPs, TIMPs and related transcription factors determine the progression of MF [21]. Transforming growth factor-β (TGF-β) is also an important factor that regulates MMP gene expression [22]. Our results suggested that prenatal exposure to LPS resulted in increased TIMP-2, but inhibited MMP2 mRNA and protein expression in rat offspring. PDTC treatment clearly ameliorated the LPS-induced alterations in MMP2 and TIMP-2 expression.

The profibrotic cytokine TGFβ is considered to be the primary inducer of the mature myofibroblast phenotype [20]. As a strong chemokine of fibroblasts, TGF-β can induce fibroblast differentiation into fibroblasts, stimulate collagen fiber connection protein and polysaccharide synthesis and promote the accumulation of intercellular substances [23,24].

In addition to its own role in fibrosis, TGF-β induces the synthesis of other cytokines, such as platelet-derived growth factor (PDGF), tumor necrosis factor α (TNF-α) and fibroblast growth factor, among others. These factors further promote the development of MF [8,25]. In this study, prenatal exposure to LPS resulted in increased TGF-β1 and TGF-β2 mRNA and protein expression in rat offspring. PDTC treatment clearly decreased these LPS-induced alterations in TGF-β1 and TGF-β2 expression. Moreover, the trend of these changes was identical to those of TIMP-2.

In summary, inflammation induced by prenatal exposure to LPS results in increased MF in adult rats. Based on the evaluation of transforming growth factor (TGF-β) in the myocardial ECM, we found that collagen and TGF-β expression in heart tissue were increased during MF in the rat offspring. Moreover, the myocardial ECM exhibited remodeling, as indicated by an imbalance in the MMP2/TIMP-2 ratio. However, the mechanisms underlying maternal LPS exposure-induced myocardial remodeling remain unclear. The exact mechanisms responsible for maternal inflammation-induced MF in offspring also remain unclear. Based on the above results, we speculate that prenatal LPS exposure disrupted the balance of the MMP2/TIMP-2 ratio in rat offspring. The above experimental results also suggested that the prevention of MF should begin during pregnancy.

## 4. Experimental Section

### 4.1. Animals Groups

Sprague Dawley (SD) rats purchased from the Animal Center of the Third Military Medical University (Chongqing, China) were mated (one female to one male; mating was confirmed based on the analysis of the vaginal plug and a vaginal smear). One week after they were acclimatized to our institute, the pregnant rats were randomly divided into 3 groups (*n =* 8 per group): the control group, the LPS group and the LPS + PDTC group. The pregnant rats in the control group received intraperitoneal (i.p.) injections of vehicle (NS) daily from gestational Day 8 to 14. In the LPS group, the rats received i.p. injections of 0.79 mg/kg LPS (Escherichia coli 026:B6, Sigma, St. Louis, MO, USA) on gestational Days 8, 10 and 12. The rats in the LPS + PDTC group received i.p. injections of 0.79 mg/kg LPS and 100 mg/kg PDTC (Sigma, St. Louis, MO, USA) on gestational Days 8, 10 and 12. Gestation lasted for 20 to 22 days.

The rat offspring were anesthetized at six or 16 weeks of age using chloral hydrate (350 mg/kg); then, the body weight was measured, and the chest cavity was immediately opened. The hearts were excised rapidly and placed in ice-cold saline to remove the blood. Then, the hearts were weighed. CI was calculated as the ratio of the heart weight (in mg) to the body weight (in g).

### 4.2. Histological Evaluation

The heart samples were fixed in 4% formaldehyde solution and embedded in paraffin. Sections were stained with HE for histopathological examination under a light microscope. The extent of MF was scored according to Sirius red staining. Alternatively, Masson stain was used for the evaluation of collagen fibers. The collagen volume fraction (CVF) was determined via quantitative morphometry using an automated image analysis system (ImagePro Plus, Version 6.0; Media Cybernetics, Silver Spring, MD, USA). The CVF was calculated as the area of cardiac collagen/the area of the field. 

### 4.3. Real-Time PCR

The expression levels of the mRNA encoding TIMP-2, MMP-2, TGFβ-1 and TGFβ-2 were assessed via real-time PCR when the rat offspring were 6 or 16 weeks of age. Total RNA was extracted from the kidneys using an RNA simple Total RNA Kit (TIANGEN Biotech, Beijing, China) and then quantified by measuring the absorbance at 260 nm. Then, total RNA (1 µg) was reverse-transcribed into cDNA using a PrimeScript™ RT Reagent Kit With gDNA Eraser (TaKaRa Biotechnology, Dalian, China). GAPDH was used as an internal control. The PCR primers were designed using Premier 5.0 (PREMIER Biosoft International, Palo Alto, CA, USA) according to published nucleotide sequences. The sequences of the primers used in this study are presented in Table 1. Each real-time PCR reaction was conducted in a total volume of 25 µL containing SYBR^®^ Premix Ex Taq™ II (Tli RNaseH Plus) (TaKaRa Biotechnology, Dalian, China) in an Eppendorf MasterCycler ep realplex system (Eppendorf, Hamburg, Germany) under the following conditions: 30 s at 95 °C followed by 40 cycles at 95 °C for 15 s, 60 °C for 15 s and 72 °C for 20 s. After amplification, melting curve analysis was performed by collecting fluorescence data while increasing the temperature from 65 to 99 °C over a period of 135 s. The relative expression ratio of each mRNA was calculated using the formula 1/2^ΔΔ*C*t^.

**Table 1:** Sequence of oligonucleotides used as PCR primers.

Gene	Position	Primer Sequences
TIMP-2	Up	5'GAATATCTAATTGCAGGGAAGGC3'
	Down	5'TGGGTGATGCTAAGCGTGTC3'
MMP-2	Up	5'AGCTGGCCCTGTTCTGACG3'
	Down	5'CACCCTCTTAAATCTGAAATCACC3'
TGF-β1	Up	5'GGCGGTGCTAAGCGTGTC3'
	Down	5'TGTTGCGGTCCACCATTAGC3'
TGF-β2	Up	5'TGTGAGAAGCCGCAGGAAGT3'
	Down	5'CAGAGTGAAGCCGCAGGAAGT3'

### 4.4. Western Blot

Total protein was extracted from the adipose tissue of 6- and 16-week-old rat offspring, and the protein concentrations were measured using the bicinchoninic acid (BCA) method. After denaturation and electrophoresis on sodium dodecyl sulfate (SDS)-polyacrylamide gels, the separated proteins were transferred to nitrocellulose membranes. Then, the membranes were blocked in 5% nonfat milk in Tris-buffered saline and Tween 20 (TBST) for 1 h. After incubation in primary antibodies (anti-TIMP-2 (Abcam, Cambridge, U.K.), anti-MMP-2 (Abcam, Cambridge, U.K.), anti-TGFβ-1 and anti-TGFβ-2 (Santa Cruz Biotechnology, Santa Cruz, CA, USA) or GAPDH (Sigma, St. Louis, MO, USA)) in TBS at 4 °C overnight, the membranes were incubated in a peroxidase-conjugated secondary antibody in TBS at room temperature for 1 h. Specific bands were detected via a chemiluminescence assay and recorded on X-ray film. Quantity One software (Bio-Rad, Hercules, CA, USA) was used to quantify the band intensities.

### 4.5. Statistics Analysis

All of the data are expressed as the means ± SEM and were analyzed using the SPSS 13.0 software package (SPSS, Chicago, IL, USA). Comparisons between the groups were performed using one-way ANOVA followed by Fisher’s least significant difference (LSD) *post hoc* test. *p* < 0.05 was considered to be significant; *p* < 0.01 was considered to be significant difference.
